# Differences in personal and lifestyle characteristics among Zimbabwean high school adolescents with and without recurrent non-specific low back pain: a two part cross-sectional study

**DOI:** 10.1186/s40945-015-0014-9

**Published:** 2015-12-01

**Authors:** Matthew Chiwaridzo, Nirmala Naidoo

**Affiliations:** 1 Department of Rehabilitation, University of Zimbabwe, College of Health Sciences, P.O Box A178, Avondale, Harare Zimbabwe; 2 Division of Physiotherapy, School of Health and Rehabilitation Sciences, University of Cape Town, Faculty of Health Sciences, Cape Town, South Africa

**Keywords:** Adolescents, Risk factors, Zimbabwe, Recurrent non-specific low back pain

## Abstract

**Background:**

Recurrent non-specific low back pain (NSLBP) is increasingly becoming common among adolescents worldwide. A recent study in Zimbabwe showed a relatively high prevalence (28.8 %) among high school students. Influential associated factors, however, remain unclear. This is a significant shortcoming. The aim was to determine personal or lifestyle-related factors associated with recurrent NSLBP among high school adolescents in Harare, Zimbabwe.

**Methods:**

This study was part of a large epidemiological study conducted in two continuous parts. Part one sought to determine self-reported associated factors among 532 participants (mean age =16 ± 1.72 years) drawn randomly from selected government schools using a reliable and content-validated questionnaire (Kappa coefficient, k = 0.32–1). Part two purposively identified adolescents (*N* = 64, median age =17 years, interquartile range, IQR = 15–18 years) with a history of ‘severe’ recurrent NSLBP from part one based on a specific eligibility criteria and compared body mass index, relative school bag weight and hamstring flexibility with matched adolescents without NSLBP. Data was analysed using Statistica version 11. Independent *t*-tests or χ^2^ tests of association were used for continuous and categorical data, respectively. The statistical significance was set at *p* < .05.

**Results:**

Recurrent NSLBP was associated with self-reported factors such as perceptions of a heavy school bag [χ^2^ (1) = 85.9, *p* < 0.001]. A significant proportion of adolescents with recurrent NSLBP spent over 30 min carrying the school bag to and from school [χ^2^ (1) =32.2, *p* < 0.001]. It was also associated with prolonged sitting (*p* < 0.001), not playing sports [χ^2^ (1) =5.85, *p* = 0.02] and tight hamstrings [χ^2^ (1) =7.6, *p* = 0.006].

**Conclusions:**

Although conclusions from this study are hesitant because of the cross-sectional nature of the study and the relatively small sample size in follow-up study, recurrent NSLBP is associated with perceptions of a heavy school bag, duration of school bag carriage, no sports participation, prolonged sitting on entertainment activities, and tight hamstrings. These findings add to the importance of promoting physical activity at school or home especially aimed at improving muscle flexibility.

## Background

Recurrent non-specific low back pain (NSLBP) affects 13 to 40 % of adolescents worldwide [1–4]. Lately, it has gained considerable research attention largely because of adverse consequences such as increased medical attention, school absenteeism, functional disability and chronic pain evolving into adulthood [3, 5]. A recent study conducted in Zimbabwe by Chiwaridzo and Naidoo [6] showed a high prevalence rate of recurrent NSLBP among urban high school adolescents. Unfortunately, attention and health resources in Zimbabwe have been focused mainly on preventation and treatment of adulthood low back pain. This is a cause of concern given the reported consequences of adolescent recurrent NSLBP and the inevitable potential to become a future musculoskeletal problem [5, 7].

The adolescence period represents a critical stage of spinal development characterised by rapid growth [8]. During this period, the spine is thought to be vulnerable to stresses that are common. Although many factors can lead to back pain, it is possible to hypothesise that factors contributing to increased load on the lumber spine or diminished blood supply to the area may lead to recurrent NSLBP [7–9]. Personal or lifestyle-related factors such as body mass index (BMI), muscle flexibility, sports participation, smoking, prolonged sitting, carriage of heavy school bags maybe important in the development of recurrent NSLBP in adolescents. These factors are interesting from a public health perspective since they are amenable to educational strategies. There is dearth of literature regarding the factors associated with recurrent NSLBP in Zimbabwean adolescents. This creates a vacuum of validated evidence to justify any preventative strategies. An understanding of the differences in personal and lifestyle characteristics among adolescents with and without recurrent NSLBP is crucial in guiding the development of preventative actions that are context relevant, since the background of other studies may be different to ours [10].

Although Zimbabwean adolescents are no different from the rest of world, differences that exist in the school system, school environment, families, lifestyle and background of school children makes them different from those in other countries. With the recent positive changes in the Zimbabwean economy, there has been growing concern over increased obesity, sedentary lifestyles, and social ills such as smoking among high school adolescents [11]. Additionally, a casual observation of the Zimbabwean scholars has shown that they carry school bags to school with items that include textbooks, sports equipment and laptops because of increased educational demands. These items may represent a substantial load if carried every day. This study was, therefore, conducted to determine the differences in personal or lifestyle-related factors between adolescents with recurrent NSLBP and those without in high schools in Harare, Zimbabwe. The factors selected were: body mass index, relative school bag weight, perceptions of school bag weight, duration and method of bag carriage, time spent sitting per day after school on entertainment activities, smoking, sports participation, and hamstring muscle flexibility.

## Methods

### Study design and participants

This article formed part of a large epidemiological study conducted in two continuous parts. The purpose of the first part was to determine personal or lifestyle-related factors associated with recurrent NSLBP among 532 randomly selected high school adolescents from government-administered schools between the ages of 13 and 19 years based on self-report using a validated and reliable questionnaire. The methodology of this part has been described in detail elsewhere [6]. Participants with recurrent NSLBP had to report pain which had occurred at least two times over the past year with each episode of lasting at least 24 h, with pain intensity of greater than two on the visual analogue scale (VAS) with at least a 30-day pain free period between the episodes [12].

### Instrument

The questionnaire used had four sections. Section A gathered information on the prevalence of recurrent NSLBP. Section B sought information related to the use of school-bags. Respondents were specifically asked questions on school bag use, perceptions of the school bag weight, duration of carriage and method of carriage. Section C captured information regarding sports participation either at school or home and the amount of hours spent in sports per week. Section D sought information on the smoking status and the amount of cigarettes smoked per week. A preliminary test and re-test reliability study showed fair to perfect kappa coefficients (0.32 to 1) of the 21 primary questions in the questionnaire. The results of the reliability study have been presented elsewhere in detail [6]. In addition, the questionnaire was assessed for content validity by a panel of five experts in the field of musculoskeletal research yielding a scale-level content validity index (S-CVI) of 0.70.

### Second part of the study

The second part of the study looked into objectively-measured factors such as BMI, hamstring flexibility and relative school bag weight, in an analytical study comparing adolescents with previous reports of recurrent NSLBP and those without. Owing to feasibility constraints, it was not possible to measure all participants in the first part of the study. Therefore, adolescents with a history of recurrent NSLBP whose episodes in the last 12 months were characterised with all the following parameters of ‘severity’ were purposively-selected from the data collection sheet to be in the study:

- Moderate to severe episodes of recurrent low back pain (an average score of ≥ 5 on the Visual Analogue Scale for pain intensity).
- Accompanied with reports of radiating pain to the lower legs (sciatica)
- Interfered with daily functional activities (a score of ≥ 5 on the modified Hanover Low Back Pain Disability Questionnaire).
- Associated with at least a one-day history of consulting health-care professionals such as nurses, medical doctors, pharmacists, physiotherapists for symptoms of recurrent NSLBP.
- Adolescents without ‘point’ recurrent NSLBP on the day of the survey as this would limit their performance in some of the tests.

**Fig. 1:**
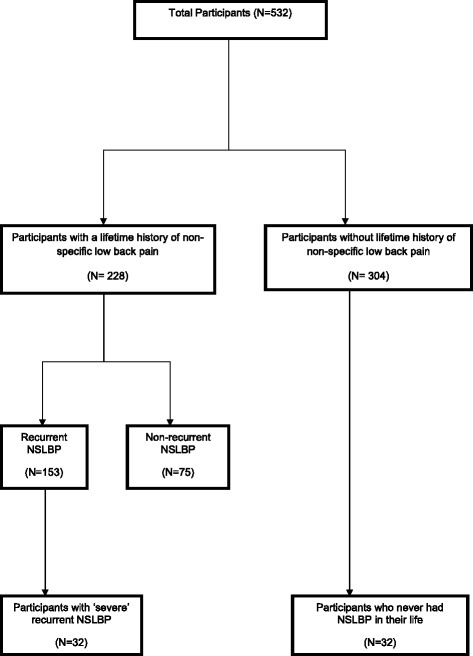
Flow chart of participants

From first part of the study, 32 adolescents were identified to be eligible (Fig. 1). These participants represented an extreme group of adolescents who experienced ‘severe ’recurrent NSLBP in the last 12 months. However, eligible adolescents who did not carry school bags to school on the day survey and whose high hairstyles did not allow accurate height measurements to be performed and were absent on survey day were excluded. To obtain a comparison sample, part one data collection sheet was analysed and school-children at the same school and in the same class matched one on one for age and gender with no history of NSLBP were selected.

### Procedure

Institutional approval was sought from selected high schools. Ethical approval for the study was obtained from the University of Cape Town, Human Research Ethical Committee (HREC/189/2012) and Medical Research Council of Zimbabwe (MRCZ/B/356). Informed consents and assents were obtained from parents and students respectively. The procedure of the first part of the study has been described elsewhere [6]. This section will describe the procedure for the second part in detail.

After the preparatory phase of visiting the selected schools consecutively explaining the study procedural issues to the school authorities and identifying eligible students, data collection was conducted on random days for two weeks in the mornings on dates agreed upon by school authorities [13]. For each student, all the measurements were conducted in a circuit fashion by the researcher in an empty classroom. However, the researcher was blinded to which group the students belonged to. In a preliminary study, inter-tester reliability testing had shown high degree of absolute agreement between the researcher and another experienced physiotherapist [Intraclass coefficient correlation, ICC = 0.95, 95 % CI = 0.94–0.96 for average flexibility measurements and ICC = 0.99, 95 % CI = 0.97–0.99 for average height measurements]

### Chair Sit and Reach test (CSR)

The procedure used for measuring hamstring flexibility using the CSR test was based on the description given in other studies [14]. Briefly, the researcher began by explaining the rationale of the CSR test and demonstrating the procedure to the student. The students were allowed to perform two demonstration trials before the actual test. Instructions to remove school hats, school blazers, school jerseys, socks, and shoes were given. A three-minute general static stretch was performed before the test. A standard school-chair stabilised against a wall was used. The instructions for the test were then given by the researcher as described in Baltaci et al. [14]. To ensure accuracy, two measurements to the nearest 0.1 cm were taken for both legs to calculate the average score. The leg yielding the best result determined the CSR score for the student.

### Anthropometric measures

Body weight was measured using a calibrated scale following standard procedures. The weight was considered as the average of two best measurements that agreed within 0.1 kg. Subsequently, each participant was weighed whilst carrying their school bag. The school-bags were weighed without assessing for the contents of the bag. The participating students were blinded to the fact that the study was about the relationship between recurrent NSLBP and school-bag weight. Students were told that the study was about “standing posture assessed when carrying a school bag” with no other information provided. This was done to capture the typical weight of the school bags and to avoid tampering of the school bags by students. Standing height was measured to the nearest 0.1 cm using a tape measure following instructions described in literature [8].

### Statistical analysis

Statistical analysis was performed using STATISTICA version 11. Kolmogorov Smirnov and Lilliefors tests were used to assess for normality of data for the first part of study whilst Shapiro Wilk test was used for the second part of study. Descriptive statistics, including mean with standard deviation was used to describe normally distributed continuous data, and the median with the interquartile range for skewed data. Independent *t*-tests or χ^2^ tests of association were used for continuous and categorical data, respectively. The Fisher’s exact test replaced the Chi-square test in 2 × 2 tables when the expected frequency in any of the cells was < 5. The statistical significance was set at *p* < .05. BMI was calculated as mass divided by the square of height (kg/m^2^) and classified based on age and gender-specific percentile values: underweight (<5^th^ percentile), normal weight (5^th^- < 85^th^ percentile), overweight (85^th^- < 95^th^ percentile) and obese (≥95^th^ percentile) [15].

## Results

### Part one study

**Fig. 2:**
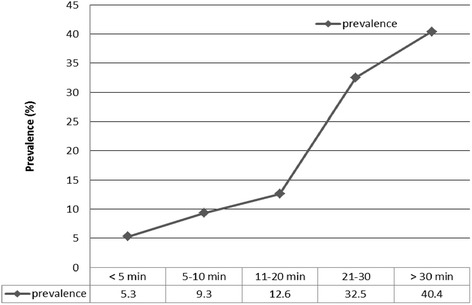
Prevalence of recurrent non-specific low back pain by duration of school bag carriage

The baseline characteristics of part one participants have been presented elsewhere [6]. The response rate for the study was 97.8 %. The data on self-reported personal and lifestyle-related factors are presented on Table 1. The majority of the school-children (98.7 %, *n* = 525) used school bags. Recurrent NSLBP was significantly related to perceptions of a heavy school bag [*χ* ^*2*^ (1) =85.9, *p* < 0.001] and duration of carriage [*χ* ^*2*^ (1) =32.2, *p* < 0.001]. The prevalence of recurrent NSLBP increased with duration of carrying the school-bag (Fig. 2). There was no association found between method of carrying the school-bag and recurrent NSLBP. Recurrent NSLBP was significantly associated with not playing sports at all or playing for 0–2 h per week. Recurrent NSLBP was not related to type of sport. There was no significant association found between smoking (current) and recurrent NSLBP. However, recurrent NSLBP was significantly associated with report of spending between five to six hours per day sitting (*p* < 0.001).

**Table 1:** Self-reported associated factors for recurrent non-specific low back pain (*n* = 532)

Variable	LBP (%)	No LBP (%)	*Χ* ^*2*^ *(1)*	*P* value
School bag use				
Yes	151 (98.7)	374 (98.7)	0.00	0.99
No	2 (1.3)	5 (1.3)		
Total	153	379		
Perceptions of bag weight				
Average	60 (39.7)	153 (40.9)	0.06	0.80
Heavy	72 (47.7)	41 (11)	85.9	<0.001
Light	19 (12.6)	180 (48.1)	57.7	<0.001
Total	151	374		
Duration of carriage				
<5 min	8 (5.3)	77 (20.6)	18.5	<0.001
5–10 min	14 (9.3)	81 (21.7)	11.1	<0.001
11–20 min	19 (12.6)	74 (19.8)	3.83	0.05
21–30 min	49 (32.5)	78 (20.9)	5.70	0.02
>30 min	61 (40.4)	64 (17.1)	32.2	<0.001
Total	151	374		
Method of carriage				
Hands	27 (17.9)	74 (19.8)	0.25	0.62
Back	68 (45)	152 (40.6)	0.85	0.36
Shoulders	56 (37.1)	148 (39.6)	0.28	0.60
Total	151	374		
Sports participation				
Yes	50 (32.7)	167 (44.1)	5.85	0.02
No	103 (63.7)	212 (55.1)		
Total	153	379		
Weekly sport duration				
<2 hrs	24 (48)	16 (9.6)	37.8	<0.001
2–4 hrs	14 (28)	36 (21.6)	0.9	0.34
5–6 hrs	8 (16)	55 (32.9)	5.36	0.02
7–10 hrs	2 (4)	55 (32.9)		<0.001*
>10 hrs	2 (4)	5 (3.0)		0.66*
Total	50	167		
Current smokers				
Yes	4 (2.6)	13 (3.4)	0.23	0.63
No	149 (97.4)	366 (96.6)		
Total	153	379		
Time spent sitting/day				
<2 hrs	14 (9.2)	61 (16.1)	4.34	0.04
3–4 hrs	47 (30.7)	23 (60.9)	39.9	<0.001
5–6 hrs	70 (45.8)	43 (11.3)	77.1	<0.001
7–10 hrs	20 (13.1)	33 (8.7)	2.32	0.13
>10 hrs	2 (1.3)	11 (2.9)	1.16	0.28
Total	153	379		

*Fisher exact two tailed test

### Part two study

Table 2 summarises the baseline characteristics of part two study participants. The sample consisted of 64 school-children. The response rate for the study was 100 %. The age distribution of the participants was not normally distributed as indicated by Shapiro Wilk test [W = 0.93, *p* < 0.01]. The median age of the participants was 17 years. In terms of gender, there were 26 males (13 cases vs. 13 controls) and 38 females (19 cases vs. 19 controls). There were no significant differences in mean body weight and height between the groups. Table 3 shows the relation between objectively-measured risk factors and recurrent NSLBP. The average BMI of the school-children was 24.8 (SD =3.02) kg/m^2^. The mean BMI for adolescents with recurrent NSLBP was 24.2 (SD = 2.88) kg/m^2^ compared to 25.2 (SD = 3.07) kg/m^2^ for adolescents without recurrent NSLBP. This difference was not statistically significant [t (62) = 1.65, *p* = 0.10]. BMI was not associated with recurrent NSLBP. The weight data for the school-bags followed a normal distribution [*W* = 0.98*, p* = 0.59] with a mean weight of 5.02 kg (SD = 0.93, range 2.5–7.4 kg). However, the mean school-bag weight for adolescents with recurrent NSLBP was significantly higher compared to adolescents without recurrent NSLBP [t (62) = −2.16, *p* = 0.03]. The mean relative school-bag weight was 8.1 % (SD = 1.91, range 3.9–13.1). However, there was no significant difference between the groups [t (62) = −1.55, *p* = 0.13]. The relative school-bag weight was dichotomised into two categories: heavy school-bags (≥10 % of body weight) and light (<10 % of body weight). There was no significant association between the groups (*p* = 0.11). The mean CSR score for all participants was −0.70 cm (SD = 3.32, range −8 to 5 cm). Between the groups, there was a significant difference in the mean CSR score [t (62) = 3.18, *p* = 0.002]. The CSR scores were dichotomised as low flexibility (CSR < 0 cm) and high flexibility (CSR ≥ 0 cm). The majority of cases (*n* = 22) had negative CSR scores [χ^2^ (1) = 7.57, *p* = 0.006]

**Table 2:** Baseline, anthropometric and muscle flexibility characteristics for part two participants (*n* = 64)

Characteristics	Participants (*n* = 64)
Age (median, IQR^a^) years	17 (15–18)
Gender (male/female)	26/38
Years of education (mean, SD)	11 (1.61)
Weight (mean, SD^b^) kg	62.5 (6.39)
Standing height (mean, SD) cm	158.9 (10.3)
BMI (mean, SD)	24.8 (3.02)
CSR (mean, SD) cm	- 0.7 (3.32)

^a^IQR- Interquartile range

^b^SD- Standard deviation

**Table 3:** Comparisons of the adolescents with recurrent non-specific low back pain and those without on relative school bag weight, BMI and CSR scores

Variable	Outcome measure	LBP	No LBP	Statistic	*p* value
BMI (kg/m^2^)	Mean (SD)^a^	24.8 (2.88)	25.2 (3.07)	t(62) = 1.65	0.10
School bag weight/kg	Mean (SD)	5.27 (0.92)	4.78 (0.89)	t(62) = −2.16	0.03
Relative school bag weight (%)	Mean (SD)	8.51 (2.08)	7.78 (1.69)	t(62) = −1.55	0.13
School bag weight (%)	<10 % ^b^BW (light)	23 (71.9)	29 (90.6)		0.11*
	≥10 % BW (heavy)	9 (29.1)	3 (9.40		
Relative school bag weight	Mean (SD)	8.51 (2.08)	7.77 (1.69)	t(62) = −1.55	0.13
CSR scores (cm)	<0 (inflexible)	22 (68.8)	11 (34.4)	Χ^2^ (1) = 7.6	0.006
	≥0 (flexible)	10 (31.2)	21 (65.6)		
CSR scores (cm)	Mean (SD)	−1.91 (3.47)	0.56 (2.69)	t(62) = 3.18	0.002

*Fishers exact test, ^a^Standard deviation, ^b^Body weight

.

## Discussion

The present study showed that most Zimbabwean high school adolescents use school bags which they carry mostly over both shoulders; a finding consistent with other reports [16–19]. However, recurrent NSLBP was significantly associated with time spent carrying the school-bag. This finding corresponds with reports of other studies [20, 21]. The fact that the association was found over 20 min suggests that recurrent NSLBP might occur after a certain critical point of carrying the school-bag. Our findings reveal a major concern for the Zimbabwean scholars who carry school bags every day and spend time carrying the bag. Further studies are, however, needed to examine the influence of other factors such as physical fitness which may contribute to the risk of injury [22].

Perceptions on school bag weight provide a proxy measure of the actual school bag weight and are reflective of muscle strength, endurance and control [16, 21]. In the present study, a significant proportion of adolescents with recurrent NSLBP perceived the school-bags to be heavy. Szpalski et al. [23] found similar results that school-children who responded positively to the question “do you find your satchel too heavy?” reported NSLBP. However, the present study showed no significant difference in the relative school bag weight between school-children with recurrent NSLBP and those without. The overall relative school bag weight of the participants was 8.1 %; a finding similar to study results by Chang et al. [20]. This is less than the global standard of 10 % proposed as a risk factor for recurrent NSLBP [20]. A plausible explanation could be the relatively low school-bag weights carried by the adolescents in the present study with a mean of 5.02 kg. The mean weight of school-bags in our study was lighter than 8.3 kg found by Sheir-Neiss et al. [18]. However, in the present study, the weight of the school-bags may have been under-estimated as they were weighed only. A single measurement may not have accurately captured the actual school bag weight. Previous studies report substantial variability in school bag weight depending on the day of the measurement [7, 24]. Our data provide a cautious support for the 10 % cut off point for relative school-bag weight for school-children, but future studies are needed to determine an appropriate cut-off point specific for the Zimbabwean population of school-children carrying school-bags.

There is mixed evidence supporting sports as a contributor or a protective agent for back pain in adolescents in the literature [23, 25–27]. For the present study, playing any form of sport for 4–10 h per week was found to be protective for recurrent NSBLP. This was so despite reduced level of sports participation found in the study of only 40.8 % adolescents. This creates a need to advocate for more sports participation in Zimbabwean schools. However, due to the nature of this study, it is not clear if sport participation “caused” recurrent NSLBP or pre-existing symptoms of NSLBP were exacerbated by sport participation resulting in school-children avoiding taking part. Furthermore, the present study showed that recurrent NSLBP was significantly associated with the report of spending five to six hours seated per day on entertainment activities. These findings add support to the conclusions of other authors who reported that adolescents are prone to low back pain because of the time spent engaged in sedentary activities [18, 22, 28, 29]. These findings add to the importance of promoting physical activity at home. At home, parents may be encouraged to create a platform for exercises as a family habit and make an active commitment to dissuade prolonged sitting.

Low hamstring flexibility could be an important individual risk factor for the development of recurrent NSLBP in adolescents. This was indicated by a significantly higher mean CSR score for the controls. These finding supports previous research [2, 15, 30]. The CSR test was employed in the present study because of cultural reasons. Zimbabwean female students wear skirts (slightly above the knee) thus making it difficult for traditional tests such as straight-leg raise (SLR) to be used. Muscle flexibility has been reported to reduce injury risk [31]. This protection is probably reduced with low flexibility. However, it is also possible that recurrent NSLBP could have had an effect on hamstring flexibility resulting in reduced CSR scores.

### Limitations

Conclusions from this study should be drawn cautiously because of the cross-sectional nature of the study and the relatively small sample size used in the second part of the study. Therefore, it is only possible to deduce a hypothesis of association between recurrent NSLBP and the identified factors. The usual limitations of recall bias and forward telescoping typical of prevalence studies threaten the reliability of the results [32]. NSLBP is complex multifactorial symptom; this study could have missed on the potential interactions of many factors in the development of recurrent symptoms among adolescents. Nevertheless, this study provides baseline information crucial in informing school policies on adolescent health and in directing future researches. Future studies may need to have larger samples to increase the power of the study.

## Conclusion

Recurrent NSLBP is a common health problem among adolescents in high school. It is associated with perceptions of a heavy school bag, duration of school bag carriage, no or little sports participation, increased hours sitting on entertainment activities, and tight hamstrings. These findings add to the importance of promoting physical activity at school or home especially aimed at improving muscle flexibility. Concerted efforts are needed from parents and teachers to restrict prolonged sitting in class or home. Schools can schedule exercise programmes throughout the school day creating opportunities for students to be active between classes. At home, parents may be encouraged to create a platform for exercises as a family habit and make an active commitment to dissuade prolonged sitting. Students should be educated on the possible dangers of carrying heavy school bags and, if possible, appropriate measures that restrict prolonged carrying of school bags such as the use of lockers can be introduced in schools.

## Abbreviations

BMI: Body mass index
BW: Body weight
CI: Confidence Interval
CSR: Chair Sit and Reach
ICC: Intra class correlation coefficient
LBP: Low back pain
IQR: Interquartile range
NSLBP: Non-specific low back pain
SD: Standard deviation
SLR: Straight Leg Raise
VAS: Visual Analogue Scale
